# Successful treatment of prolonged COVID-19 with remdesivir and nirmatrelvir/ritonavir in a patient with a history of diffuse large B-cell lymphoma: a case report

**DOI:** 10.1186/s43046-025-00291-1

**Published:** 2025-06-30

**Authors:** Nadia Bouhamdani, Dominique Bouhamdani, Cynthia Léger, Josiane Stadler, Nancy Saulnier

**Affiliations:** 1 Vitalité Health Network, Moncton, Canada; 2 Atlantic Cancer Research Institute, Moncton, Canada; 3 Université de Sherbrooke, Sherbrooke, Canada; 4 Centre de Formation Médicale du Nouveau-Brunswick, Moncton, Canada

**Keywords:** COVID- 19, SARS-CoV- 2, Hematological malignancies, Diffuse large B-cell lymphoma, Remdesivir, Veklury, Nirmatrelvir/ritonavir, Paxlovid

## Abstract

**Background:**

Immunocompromised individuals, such as those affected by and treated for hematological malignancies, face a higher risk of prolonged SARS-CoV-2 infection. Increased disease risk is further compounded by limited treatment options. Currently, approved antiviral monotherapies against COVID-19 include remdesivir (Veklury) and nirmatrelvir/ritonavir (Paxlovid) which have stringent recommended prescribing windows within 7 and 5 days of symptom onset, respectively. Furthermore, these two antiviral therapies are approved for treatment lengths of 3 (remdesivir) and 5 days (Paxlovid).

**Case presentation:**

Herein, we describe the successful treatment of prolonged COVID-19 in a patient with a history of diffuse large B-cell lymphoma with an extended combination therapy; remdesivir and nirmatrelvir/ritonavir. The patient presented with symptomatic COVID-19 that was unsuccessfully treated with a 10-day course of remdesivir. After 2 months of symptomatic infection, the patient was treated with remdesivir in combination with nirmatrelvir/ritonavir for 10 days, which quickly resolved the cough and cleared viral load.

**Conclusion:**

Our case highlights the efficacy of administrating a combination treatment of remdesivir and nirmatrelvir/ritonavir outside recommended guidelines for the treatment of persistent COVID-19 infection in an immunocompromised individual. High-quality studies evaluating the usefulness of this combinatory therapy as a longer-course treatment in patients with neoplasms is warranted.

## Background

Immunocompromised individuals are at an increased risk of persistent and protracted severe acute respiratory syndrome coronavirus 2 (SARS-CoV-2) infection and coronavirus disease 2019 (COVID-19) [1]. Notably, those suffering from hematological malignancies present a disturbance in the number and function of various immune cells which increase their susceptibility to COVID-19. Many lymphoma treatments can also cause severe and long-lasting immunosuppression [2]. Thus, hematological malignancies and their treatment can inhibit the clearance of viral load and prolong infection [2]. In patients with B-cell lymphoid malignancies, SARS-CoV-2 infection has been documented to last several months [2]. Hospitalization and mortality rates are significantly higher for these individuals when compared to the general population [3–6].

Antiviral agents have proven to be an indispensable tool in treating patients at risk of severe COVID-19. Among these agents, remdesivir (Veklury) and nirmatrelvir/ritonavir (Paxlovid) have proven to be effective monotherapies [7]. Health Canada has approved both these agents for the treatment of patients with mild to moderate COVID-19 presenting with high risk of COVID-19 complications [8]. However, the administration of nirmatrelvir/ritonavir and/or remdesivir may be restrictive when looking at those who are immunocompromised. There is currently a lack of high-quality data showing that these agents can be effective in this population when administrating beyond the recommended window and length of time. In fact, remdesivir is to be administered daily for 5 days and may be extended up to a total of 10 days in patients admitted to the hospital who have pneumonia and in need of extra oxygen; patients who are not hospitalized but are at increased risk for progressing to severe COVID-19 receive remdesivir for 3 days. As for nirmatrelvir/ritonavir, recommended treatment length is 5 days. Notwithstanding, case reports have highlighted the effectiveness of short courses of remdesivir, principally in combinatory treatments, in protracted COVID-19 [9–12], and longer courses of remdesivir and nirmatrelvir/ritonavir have also successfully treated protracted COVID-19 in immunocompromised individuals [13, 14].

Herein, we describe the successful treatment of persistent COVID-19 in an immunocompromised patient with a history of diffuse large B-cell lymphoma with a prolonged combinatory treatment of remdesivir and nirmatrelvir/ritonavir.

## Case presentation

An 81-year-old male was diagnosed with stage IVB diffuse large B-cell lymphoma in March of 2020. At this time, the patient underwent a successful R-CHOP treatment with intrathecal methotrexate and was in remission until February of 2022. Cervical relapse was treated with an ineffective course of R-GEMOX up to September of the same year; after which, the patient underwent 5 courses of radiotherapy while awaiting CAR T cell treatment administered in December 2022. CAR T cell treatment proved to be efficacious, and patient is still presently in remission. Notably, patient had progressively resumed physical activity and was feeling healthy.

**Fig. 1:**
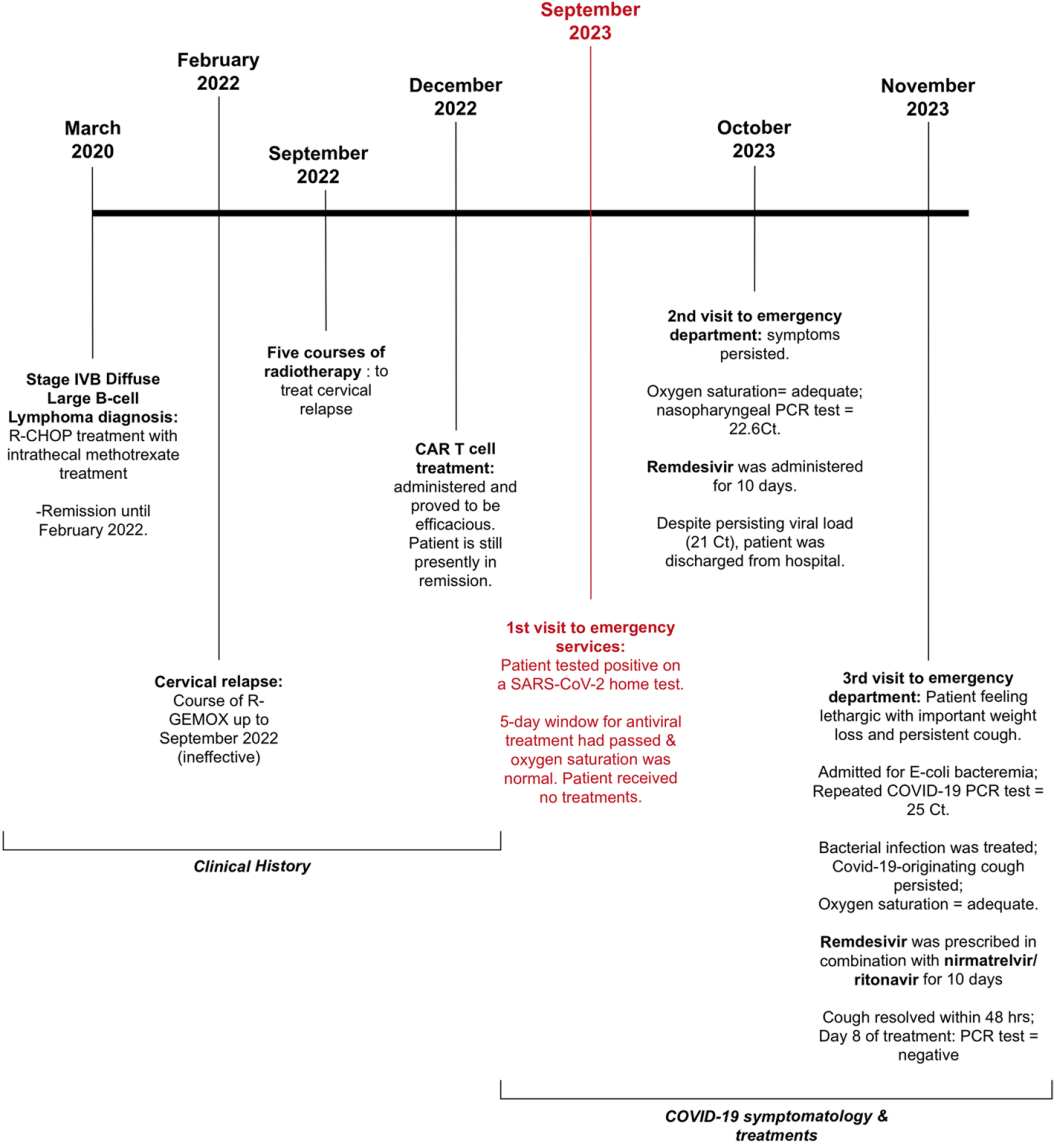
Patient timeline: detailed timeline including clinical history from March 2020 to September 2023 where patient first tested positive for COVID-19; COVID-19 symptomatology and treatments from September 2023 to November 2023

The patient presented to emergency services on September 14th 2023, reporting fatigue, cough, loss of appetite; he had tested positive on a SARS-CoV-2 home test (Point of Care Test) on September 8th. Patient was fully vaccinated. As the 5-day window for an antiviral treatment had passed and oxygen saturation was normal, no antivirals were prescribed at this point in time. Patient presented back at emergency room on October 18th; symptoms persisted, and the patient was nearly bedridden. Oxygen saturation remained adequate at this time and nasopharyngeal PCR test targeting the envelope (E) protein of the virus stood at 22.6 Ct (cycle threshold). Briefly, Ct values indicate the number of cycles required to detect viral RNA and thus, reflects the concentration of the virus in a sample. Lower Ct values indicate higher viral loads whereas higher Ct cycles indicate lower viral loads, as more cycles are needed to amplify the RNA. Patient was administered remdesivir for 10 days. Although the viral load persisted (21 Ct), patient reported feeling better and thus was discharged from the hospital. Patient presented again to emergency room on November 16th, reporting feeling lethargic, important weight loss and persistent cough. He was admitted for *Escherichia coli* bacteremia and PCR COVID-19 test was repeated which stood at Ct 25. Patient was treated for the bacterial infection, and the COVID-19 originating cough persisted albeit with an adequate oxygen saturation. At this time remdesivir was prescribed in combination with nirmatrelvir/ritonavir for 10 days. With this treatment, the cough had resolved within 48 h and a PCR test came back negative on day 8 of treatment. On follow-up appointments several months following discharge, the patient remained well and asymptomatic. A detailed patient timeline is presented in Fig. 1.

## Discussion

Herein, we present a patient with prolonged COVID-19 successfully treated with a 10-day combinatory course of remdesivir and nirmatrelvir/ritonavir. More specifically, patient did not respond to an earlier trial of remdesivir treatment; however, the use of remdesivir and nirmatrelvir/ritonavir quickly resolved symptoms and cleared the viral load. This case as well as other documented case reports have shown that the combination of remdesivir and nirmatrelvir/ritonavir can be effective past the recommended prescribing length [15]. Treatment algorithms for patients with COVID-19 have been established for hospitalized and non-hospitalized adults; however, immunocompromised patients have been left out of many studies [16]. Health Canada currently recommends treating immunocompromised patients following the same algorithm as the general population, falling into the category of patients who are at high risk of progressing to a severe manifestation of COVID-19; however, these recommendations are based solely on expert opinion as data is currently limited [17]. A randomized controlled trial found that remdesivir reduces the risk of hospitalization and death related to COVID-19, although only 14 of the 279 remdesivir patients and 9 of the 283 controls were immunocompromised patients. It is therefore unclear if remdesivir is the optimal treatment for immunocompromised patients [18]. A cohort study found a statistically significant reduction of hospitalization and death related to COVID-19 in the immunocompromised population who had received their COVID-19 vaccination when treated with nirmatrelvir/ritonavir compared to patients who had not received remdesivir [19]. While guidelines suggest monotherapy for severe COVID-19 infections, we believe that combinatory treatment of remdesivir and nirmatrelvir/ritonavir may be highly advantageous in immunocompromised patients. Some studies have found that the combination of remdesivir with other drugs can increase mortality; however, the specific combination of remdesivir and nirmatrelvir/ritonavir has not been extensively studied [20]. A recent in vitro study has, however, shown synergistic activity when using remdesivir and nirmatrelvir against SARS-Cov-2, suggesting that this combination may have a global impact on difficult-to-treat COVID-19, especially in immunocompromised patients [21]. While a 2024 target trial emulation study did not show that a nirmatrelvir/ritonavir and remdesivir combination therapy was beneficial in the general population compared to monotherapies, this was not evaluated in immunocompromised individuals [22]. In fact, a randomized clinical trial is currently underway [23].

This case report adds to previously documented cases of efficacious treatment of persistent COVID-19 with remdesivir and nirmatrelvir/ritonavir in patients with hematological malignancies [13, 14]. In fact, Pasquiniet al. documents 14 cases where an immunocompromised patient with a history of B-cell malignancies were successfully treated for their SARS-CoV-2 infection with a combination of remdesivir and nirmatrelvir/ritonavir [24]. Therefore, the combination of remdesivir and nirmatrelvir/ritonavir warrants high-quality studies evaluating its usefulness as a longer-course treatment in patients with neoplasms as data regarding treatments in this population are primarily from case reports and case series [1].

## Conclusion

This case report highlights the successful treatment of persistent COVID-19 in an immunocompromised patient with a combination of remdesivir and nirmatrelvir/ritonavir over a 10-day period. Despite the patient’s lack of response to previous treatments, this combinatory therapy effectively resolved symptoms and cleared the viral load. The findings suggest that longer courses of remdesivir and nirmatrelvir/ritonavir may be beneficial for immunocompromised patients, especially those with hematological malignancies. This case adds to existing evidence supporting the need for high-quality studies to evaluate the effectiveness and safety of this combination therapy in such populations.

## Data Availability

All relevant clinical data was included in the manuscript. No datasets were generated or analysed during the current study.

## Abbreviations

SARS-CoV- 2: Severe Acute respiratory syndrome coronavirus 2
COVID- 19: Coronavirus disease 2019
PCR: Polymerize chain reaction
